# Supplementary material for: Energy Allocation of the Wolf Spider Pardosa pseudoannulata under Dietary Restriction
Source: Insects. 2023 Jun 25;14(7):579. doi: 10.3390/insects14070579 (PMC10380717; doi:10.3390/insects14070579)
Supplement: Supplementary file 1 [file insects-14-00579-s001.zip › insects-2427924-supplementary.pdf]

Supplementary Tables

Supplementary Table S1. Number of *Drosophila melanogaster* supplied to *Pardosa pseudoannulata* in each group.

Instar	H	M	L
2nd	4	2	1
3rd	6	3	1
4th	9	5	3
5th	13	7	3
6th	20	10	5
7th till mature	20	10	5

H, high feeding; M, medium dietary restriction; L, severely dietary restriction.
